# Associations between dietary fibers and gut microbiome composition in the EDIA longitudinal infant cohort

**DOI:** 10.1016/j.ajcnut.2024.11.011

**Published:** 2024-11-16

**Authors:** Marianne K Lalli, Tuuli EI Salo, Leena Hakola, Mikael Knip, Suvi M Virtanen, Tommi Vatanen

**Affiliations:** 1 Institute of Biotechnology, Helsinki Institute of Life Science, University of Helsinki, Helsinki, Finland; 2 Department of Public Health, Finnish Institute for Health and Welfare, Helsinki, Finland; 3 Unit of Health Sciences, Faculty of Social Sciences, Tampere University, Tampere, Finland; 4 Tampere University Hospital, Wellbeing Services County of Pirkanmaa, Tampere, Finland; 5 Research Program for Clinical and Molecular Metabolism, Faculty of Medicine, University of Helsinki, Helsinki, Finland; 6 New Children’s Hospital, Helsinki University Hospital, Helsinki, Finland; 7 Center for Child Health Research, Tampere University and Tampere University Hospital, Tampere, Finland; 8 Department of Microbiology, Faculty of Agriculture and Forestry, University of Helsinki, Helsinki, Finland; 9 Broad Institute of MIT and Harvard, Cambridge, MA, United States; 10 Liggins Institute, University of Auckland, Auckland, New Zealand

**Keywords:** human milk, breastfeeding, complementary feeding, dietary fiber, gut microbiome, infants, human milk oligosaccharides, weaning, metagenomics, fecal metabolomics

## Abstract

**Background:**

The infant gut microbiome undergoes rapid changes in the first year of life, supporting normal development and long-term health. Although diet shapes this process, the role of fibers in complementary foods on gut microbiome maturation is poorly understood.

**Objectives:**

We explored how the transition from human milk to fibers in complementary foods shapes the taxonomic and functional maturation of the gut microbiome within the first year of life.

**Methods:**

We assessed the longitudinal and cross-sectional development of infant gut microbiomes (*N* = 68 infants) and metabolomes (*N* = 33 infants) using linear mixed models to uncover their associations to dietary fibers and their food sources. Fiber intakes were assessed with 3-d food records (months 3, 6, 9, and 12) relying on CODEX-compliant fiber fraction values, and questionnaires tracked the overall complementary food introduction. Bacterial species were identified and quantified via MetaPhlAn2 from metagenomic data, and metabolomic profiles were obtained using 4 LC-MS methods.

**Results:**

We identified 176 complementary food fiber-bacterial species associations. First plant-based fibers associated with microbiota compositions similar to breastfeeding, and further associated with aromatic amino acid-derived metabolites, including 5-hydroxyindoleacetic acid (total dietary fiber – complementary foods (g) – β = 3.50, CI: 2.48, 4.52, *P* = 6.53 × 10^–5^). Distinct fibers from different food categories showed unique associations with specific bacterial taxa. Key species, such as *Faecalibacterium prausnitznii*, associated with oat fibers (g/MJ, β = 2.18, confidence interval: 1.36, 2.84, *P* = 6.12 × 10^–6^), reflective of maturing microbial communities. Fiber intake during weaning associated with shifts in metabolite profiles, including immunomodulatory metabolites, with fiber effects observed in a source- and timing-dependent manner, implicated in gradual microbiome diversification.

**Conclusions:**

Introducing complementary dietary fibers during the weaning period supports gut microbiome diversification and stabilization. Even minor dietary variations shows significant associations with microbial taxa and functions from the onset of weaning, highlighting the importance of infant dietary recommendations that support the gut microbiome maturation during early life.

This trial was registered at clinicaltrials.gov as registration number NCT01735123.

## Introduction

The infant gut microbiome assembly starts at birth and follows a sequential developmental pattern where the availability of nutrients and the timing of solid food introduction plays a key role [[1], [2], [3], [4], [5]]. Simultaneously, microbe–host interactions contribute to immune system development and maturation [6,7], neuronal-development, and host metabolism [6,8,9]. Although undigested carbohydrate (glycans/fibers) as well as some protein and fats pass to colon, the immense diversity of glycan structures in human milk and later on in solid foods together pose unique selection pressures on the gut microbiome [8,[10], [11], [12], [13]]. Human milk oligosaccharides (HMOs), indigestible prebiotic fibers in human milk [10], provide substrates for the establishment of pioneering *Bifidobacterium* and *Bacteroides* species [14,15] with roles in immune maturation [[15], [16], [17], [18], [19]]. Despite extensive research on breastfeeding and formula feeding, there remains a lack of longitudinal studies on the effects of solid food derived fibers on infant gut microbiome maturation.

Understanding how complementary diet fibers influence gut microbiome species composition can help develop strategies that promote microbiome maturation patterns, supporting long-term infant health [3,8,12,20]. The weaning period, ∼4–6 mo of age, marks a critical diversification of the microbiome [1,4,5,20] as diet shifts from the breast milk HMOs [10] to plant-derived fibers. This dietary shift selects for bacterial taxa with complementary carbohydrate active enzymes ∖necessary for fermenting new glycans [11,13,21] and aids the establishment of an adult-like gut microbiome by ∼3–5 y of age [8,12,22,20]. Gut microbiome development relies on the networks of interacting microbial taxa that form functional communities aiding the assembly of the successive taxa [5,13,23,24]. Therefore, disturbances in bacterial nutrition sources during this critical period may have vast implications for health [8] and contribute to a generational loss of microbial diversity [1,5,[25], [26], [27]], exacerbating risk of allergies and chronic diseases.[24,27,28].

Previous large-scale gut microbiome analyses in healthy, partially breastfed infants provide a framework for examining how diet associates with the key expected microbial shifts [1,4,5,23,29]. In turn, recent advances in fiber analysis with updated [[30], [31], [32]] CODEX definitions that now include resistant starch and short-chain (<9 monomers) nondigestible oligosaccharides (NDOs), have improved the accuracy of fiber intake assessment [32]. These updated protocols better mimic small intestine digestion, improving the quantification of fiber providing as bacterial energy substrates reaching the colon [31,32].

Our study employs these latest CODEX-compliant methods alongside detailed 3-d food records to examine the longitudinal development of the infant gut microbiome and metabolome. By tracking the transition from a human milk-adapted microbiota to a more diverse and mature composition, we elucidate how dietary fibers and their whole food sources associate with microbiome maturation patterns during the first year of life.

## Methods

### EDIA cohort

**FIGURE 1:**
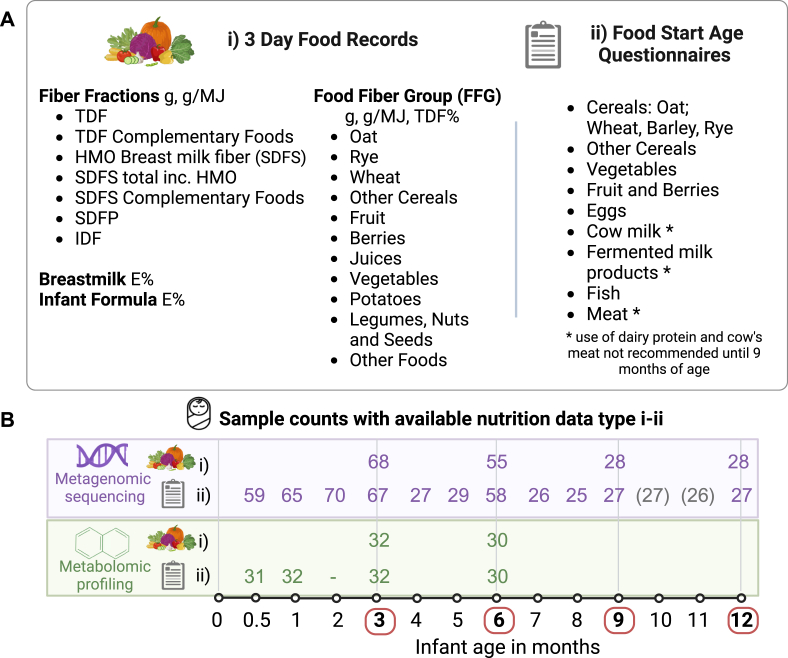
EDIA study collected metagenomic, metabolomic, and nutrition data from infants. (A) Types of dietary data included: (i) 3-d food records (quantitative data) and (ii) Food start age questionnaires (categorical, months, yes/no) with studied food categories monitored at 0.5 mo and continued at monthly intervals from months 1 to 9, concluding at 12 mo. TDF and SDFS total includes both breast milk HMO and complementary food derived fibers. The measurement categories for quantitative dietary data include absolute quantities in grams (g), energy adjusted amounts in grams per megajoule (g/MJ), and energy percentage (*E*%) for breast milk, infant formula, and macronutrients. Additionally, food sources of total dietary fiber were calculated for FFG, TDF% category describing the proportion of the food fiber source from the total food fiber intakes. “Other Foods” consists of all ingredients not included in the above FFG categories that contain fiber for example, chocolate, ketchup. “Other cereals”-category included corn, rice, buckwheat, and millet. (B) Number of samples metagenomic and metabolomic data after quality control and matched with types of dietary data (i) and (ii) at each time point are indicated. The additional samples from months 10 and 11 were used only for the following categorical variables (yes/no) with yearlong data available; overall and exclusive breastfeeding status, combined start age of any complementary solid foods and use of infant formula. Figure created with BioRender.com. EDIA, Early Dietary Intervention and Later Signs of Beta-Cell Autoimmunity; FFG, food fiber groups; SDFS dietary fiber soluble in both water and 78% aqueous ethanol; TDF, total dietary fiber.

The Early Dietary Intervention and Later Signs of β-Cell Autoimmunity (EDIA) cohort study in Tampere, Finland, followed 73 pregnant females and their newborn infants from the third trimester of pregnancy until the infant turned 1 y of age. The study recruited families during routine mid-pregnancy visits from January 28, 2013, to February 25, 2015, coinciding with fetal ultrasound screenings typically conducted around the 20th wk of pregnancy. Data collection concluded on July 26, 2016. Sample collection involved the collection of infant stool samples starting at 2 wk of age and then monthly, and were used for metagenomic and metabolomic analyses. Parental consents were obtained for sample collection and the analysis of the infant’s histocompatibility leukocyte antigen (HLA)-conferred genotype using cord blood at birth (Supplemental Figure 1). The study enrolled infants with HLA-genotypes associated with higher risk of type 1 diabetes. Exclusion criteria included prematurity (gestational age <35 wk), and severe illnesses. Within the cohort, individuals took part in a randomized controlled trial (RCT) investigating the effects of extensively hydrolyzed casein formula in infants with moderate to high genetic risk of type 1 diabetes, as previously described [33,34]. The study protocol was approved by the Ethical Committee of the Joint Municipal Authority of the Pirkanmaa Hospital District with further details available at Clinicaltrials.gov Identifier: NCT01735123. The exclusion criteria for the current study included incomplete records of nutritional or covariate information, or a missing fecal sample at a given time point (Supplemental Figure 1, Figure 1). The covariates are described in the section “Multivariable model construction". Trial design and clinical findings for RCT are described in Siljander et al. [34].

### Dietary data collection

The composition of the infant diet was monitored using 3-d weighed food records at the ages of 3, 6, 9, and 12 mo, kept by the parents and daycare providers. Mothers were encouraged to breastfeed their infants as long as possible. Infant foods containing cow milk proteins, beef, or veal were discouraged during the dietary intervention period of the first 9 mo. Additional dietary information, including the start age at which various complementary food categories were introduced (Figure 1Aand B, ii), was tracked through interviews using structured questionnaires and conducted by trained nutritionists or research nurses. These assessments began at 2 wk, continuing monthly for 1–12 mo.

The current study utilized samples from 68 infants with available metagenomic and dietary data from relevant months. Dietary variables and the exact number of metagenomic and metabolomic samples with matching nutrition data are presented in Figure 1. Fecal metabolic profiling had been performed for a subset of 33 infants (125 stool samples). The 3-d food records were reviewed by trained nutritionists. The annually updated national food composition database Fineli and associated in-house dietary calculation software tool Finessi were used to summarize the intake of energy, total dietary fiber (TDF), fiber fractions, and total fiber from various foods with and without breastmilk (Figure 1). A food composition database developed for commercial infant foods and infant formulas enabled detailed coding of these foods.

This study used the most recent available CODEX-compliant values [35,36] for dietary fiber and its fractions, which were integrated in Fineli. The Finnish Food Authority conducted direct analyses on 110 food ingredients using the CODEX-compliant, internationally approved AOAC 2011.25 method for dietary fiber quantification. Additional literature-based values allowed comprehensive update of the database, ensuring the most precise fiber content measurements available (AOAC 2009.01, 2011.25, or other) for various food ingredients [37]. The current study examined the intake and sources of TDF (including HMOs), total complementary food-related dietary fiber (TDF complementary foods), and specific dietary fiber fractions. These fractions include insoluble dietary fiber (IDF), dietary fiber soluble in water but insoluble in 78% aqueous ethanol (SDFP), and dietary fiber soluble in both water and 78% aqueous ethanol (SDFS). Food categories [Figure 1; fiber food group (FFG), food start age] were selected on the basis of their relevance to typical infant diets in Finland. Fiber intake was calculated on the basis of the specific fractions and quantities of complementary foods consumed by each infant, first accounting for variations in fiber content within food groups such as different fruits before aggregating to determine total fruit-based fiber intake per infant. This method allowed us to more precisely observe associations between fiber quantities, microbial taxa, and metabolites.

The estimated energy requirements for infants who were breastfed at 3, 6, 9, or 12 mo were calculated on the basis of their age, body weight, and the energy deposition needed for growth. These calculations followed the recommendations set by the Institute of Medicine, accounting for both daily energy needs and the energy required for healthy growth and development [38]. The amount of human milk was calculated on the basis of the difference between estimated energy requirement and energy intake reported in food records among those who were breastfed. This method is considered accurate and a practical alternative for estimating infant energy intake in studies where direct human milk measurement is not possible [39]. Dietary fiber content of human milk was based on previous literature on HMOs. This study has used the previously estimated fiber and macronutrient values typical for mature milk (120 d of lactation) considered representable for the majority of the first year [40]. Dietary fiber value registered in Fineli for human milk was 1.3 g of HMO in 100 g of human milk and energy content of 65 kcal/100 g (273 kJ/100 g), together allowing the estimation of HMO intake. HMO was classified as part of SDFS total, but intake of SDFS from complementary foods were also examined separately. The dietary calculations considered the type of infant formulas used; however, their fiber contents were negligible as the formulas used in the original RCT were not supplemented with prebiotics. Therefore, there was no difference in TDF intakes between different formula type users.

### Nutrition data analysis and statistics

Nutritional data was matched to available metagenomic and metabolomic samples and statistical analyses of the dietary intakes and their connection to microbiome features (as described below). The Wilcoxon rank-sum test (Mann–Whitney *U* test) was used for comparisons of unpaired continuous non-normally distributed nutrition and fiber intake data between groups (infants who were breastfed compared with weaned). The normality of the data was assessed visually from histograms and with the Shapiro–Wilk normality test.

### Metagenomic and metabolomic method details

#### Sample collection and DNA extractions

Mothers collected infant stool samples at home, initially freezing them at –20°C. The samples were then transferred on dry ice to the EDIA Core Laboratory in Helsinki for –80°C storage, before being sent to Tampere University for DNA extraction. Using the PowerSoil DNA Isolation Kit's vacuum protocol (MoBio Laboratories, Inc.), DNA was extracted from 0.2 g of each stool sample and subsequently preserved at –80°C.

#### Metagenome library construction, sequencing, and quality control

DNA samples were normalized to 50 pg/mL using the Quant-iT PicoGreen dsDNA Assay and prepared into Illumina sequencing libraries using the Nextera XT DNA Library Preparation kit (Illumina), with subsequent sequencing on the Illumina HiSeq 2500 platform to achieve 2.5 Gb per sample with 101 bp paired-end reads. Rigorous quality control included adaptor sequence removal (Trim Galore! v0.4.4 [41]), filtering out low-quality and human reads (KneadData v0.7.2 [42]), retaining samples with over 5 million high-quality reads for analysis. Taxonomic profiling was performed using MetaPhlAn2 [43] with a species-specific marker gene database.

#### Metabolomic analysis, MS^2^ data generation, and data processing

Metabolomic profiles of stool samples were generated using liquid chromatography-mass spectrometry (LC-MS) methods, applying 4 complementary methods including HILIC-pos ion mode MS analysis of polar metabolites, HILIC-neg for negative ion mode MS analysis of polar metabolites, C8-pos polar and nonpolar lipid analysis, and C18-neg for negative ion mode analysis of metabolites of intermediate polarity. The machinery consisted of Shimadzu Nexera X2 U-HPLC (Shimadzu Corp.) coupled to a Q Exactive Hydro Quadrupole Orbitrap or Exactive Plus Mass Spectrometer (Thermo Fisher Scientific).

Raw data processing involved TraceFinder 3.3 (Thermo Fisher Scientific) and Progenesis QI software (Nonlinear Dynamics), referencing the Human Metabolome Database (HMDB, v4.0) for metabolite identification and ClassyFire (version 1.0 [44]) for classification. Metabolomic features with matching internal standards (858) were included in the analyses. Metagenomic and metabolomic sample processing and methods have been described previously [33].

### Quantification and statistical analysis: metagenomic and metabolomic association analyses with diet using multivariable models

#### Multivariable model construction

Potential confounding factors were selected on the basis of previous infant cohort studies [5,33,45]. These studies consistently show that breastfeeding, delivery mode, infant formula use, sex (female/male), infant age (months), introduction of solid/complementary foods, and prior antibiotic use have a significant impact on infant gut microbiomes across various sampling time points during the first year of life. These factors were incorporated as fixed effects in all the statistical linear models. Models were employed to study the connection between taxa and metabolites in association with nutritional factors, the primary variables of interest. The impact of antibiotics (yes/no) was deemed significant only if taken within a month before the fecal sample, as a single course of oral antibiotics was shown to have only limited effect outside of this time window within the cohort [33].

We modeled the confounders mentioned above and age at introduction of various complementary foods (“Food Start Age”) as binary (yes/no) and categorical variables, whereas the food record data was treated as continuous values. These models were applied in all cross-sectional and longitudinal multivariate linear analyses. In longitudinal alpha diversity and species association analyses, participant identity was included as a random effect to correct for subject-specific effects caused by the repeated measurements.

#### Metagenomic associations with nutrition

Alpha diversity was measured on a species level using the Shannon index, which was calculated using the diversity function in the Vegan (version 2.5-7) R package [46]. To assess the association between alpha diversity and diet, we employed the glmmPQL function from the MASS package in R (MASS version 7.3-58.3 [47]). This approach allowed us to fit a Generalized Linear Mixed Model to the data, accounting for both fixed and random effects within the dataset [48,49]. The Shannon index significance threshold was set at nominal *P* value of 0.05.

Beta diversity was quantified using the Bray–Curtis distance metric using the Vegdist function in the Vegan R package. Bray–Curtis is a metric that considers both the presence and abundance of different taxa in the communities. The permutational multivariate analysis of variance (PERMANOVA) was employed to assess the amount of variance each variable can explain in beta diversity, that is, the Bray–Curtis distances between the infant samples, using 9999 permutations. The PERMANOVA test was implemented in adonis2 function in the vegan package (version 2.5–7) by evaluating the marginal contributions of each covariate (argument “by=margin”) where each term was fitted while considering all other terms in the model, regardless of their order or position. The PERMANOVA analyses do not permit the specification of random effects preventing longitudinal analyses. We have reported false discovery rate (FDR)-corrected *P* values (*Q*-values), FDR <0.05 considered as significant. Significant results were visualized using NMDS plots and used for descriptive purposes only. Multiple testing corrections were conducted using the p.adjust function via the Benjamini–Hochberg method.

For species level analyses, we employed a statistical tool called Multivariable Association with Linear Models, MaAsLin2 (package version 1.7.2) [50], which is designed to identify associations between microbiome profiles—taxonomic, functional, or metabolomic—and complex metadata such as nutrition with multiple additional fixed effects, that is, confounders and random effects caused by repeated measurements. We used log-transformed species-level relative abundances in a linear multivariate model, where dietary components were the main variables considered along with covariates. In the MaAsLin2 analysis, a common taxonomic prevalence filter was applied at 10% and the significance threshold was set at an FDR-corrected *P* value of 0.25, common for explorative analyses, unless stated otherwise.

Specifically, all the longitudinal and cross-sectional nutritional analyses for *1*) alpha diversity, *2*) beta diversity (cross-sectional only), and *3*) per taxa analyses were based on the mean absolute and energy adjusted values of continuous nutritional metadata variables obtained from 3-d food records collected at months 3, 6, 9, and 12 (Figure 1A). For categorical binary (yes/no) variables including breastfeeding status, exclusive breastfeeding, and the first introduction of any solid foods in the diet, data from 0.5 month and monthly intervals from 1 to 12 months were used for longitudinal analyses, with associations determined over time. The introduction age of various solid food categories (as described on nutritional data, “Start age questionnaire”) allowed longitudinal analyses from 2 wk of age and continued at monthly intervals from months 1 to 9, concluding at 12 mo. Additionally, the impact of nutritional variables was investigated cross-sectionally at each month 3, 6, 9, and 12, in a linear effect model therefore omitting the age and participant ID from the models (1 sample/infant). Beta diversity was assessed cross-sectionally as above for both food record data and the age at introduction of various food categories (Food Start Age, Figure 1).

#### Metabolic associations with nutrition

Metabolomic profiling was carried out for a subset of 33 infants, and included months 0.5, 1, 3, and 6, therefore, limiting the nutritional analyses for these corresponding months. Otherwise, similarly to longitudinal taxonomic analyses, we assessed the influence of nutrition on fecal metabolites by employing the MaAsLin2 package (version 1.7.2), utilizing the base of the constructed linear mixed effect model. Within these models, we treated metabolic products as the outcomes of interest, whereas dietary components were the main variables considered along with the common covariates and subject ID as random effects.

As described above, the longitudinal nutritional analyses were based on the mean absolute and energy adjusted values of continuous nutritional metadata variables obtained from 3-d food records and monthly food questionnaires. In the MaAsLin2 analysis, a common metabolite prevalence filter was applied at 10% and the significance threshold was set at an FDR-corrected *P* value of 0.25, common for explorative analyses, unless stated otherwise.

#### Data analysis tools

All data processing and statistical analyses, for both metagenomic and metabolomic data with metadata, were performed using R version 4.3.0 (2023-04-21) and available packages such as tidyr (v1.3.0). Figure panels and other visualizations were created using ggplot2 (v.3.3.5), whereas species-level association heatmaps were created using pheatmap (v1.0.12), all of which are distributed through CRAN, part of the R Foundation for Statistical Computing. Missing intensity values of metabolomic data were imputed with half of the minimum value per metabolomic feature, before the statistical analyses.

## Results

### Key characteristics of the cohort

The study included 64 infants with completed food records and fecal samples (68 for food start age questionnaires) with a decline in sample numbers across age groups: 64 at mo 3, 55 at month 6, and 27 at months 9 and 12 (Figure 1). Seven (10.9%) infants were delivered via cesarean section. Antibiotic use was low and unrelated to breastfeeding status. Cohort characteristics relevant to gut microbiome remained consistent despite participant dropouts, with 7.4% (2/27 infants) cesarean delivery rate at months 9 and 12 (Supplemental Table 1). The fecal microbiomes were characterized using metagenomic sequencing followed by species-level taxonomic profiling and untargeted fecal metabolomics were completed by 4 complementary LC-MS methods until 6 mo of age (Figure 1).

### Breastfeeding status was the main determinant for overall fiber and macronutrient composition of the infant diet during the first year of life

**FIGURE 2:**
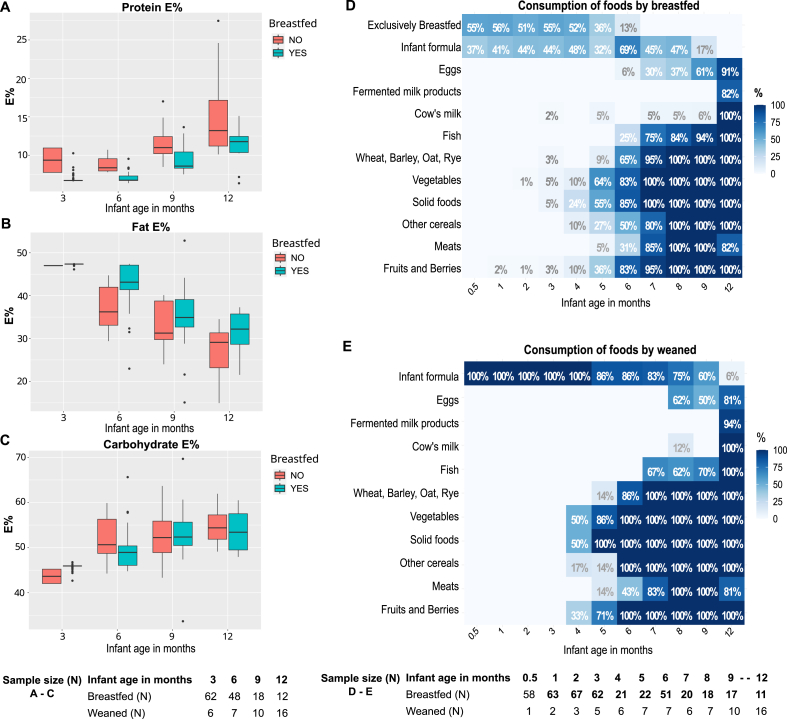
Macronutrient intakes and consumption of different foods by breastfeeding status during first year of life. Percentage of energy obtained from (A) protein (*P* < 0.05 across all months), (B) fat (*P* < 0.05 months 3, 6, and 12), and (C) carbohydrates (*P* < 0.05 month 3), stratified by breastfeeding status. For boxplots, midlines represent the median, boxes the IQR (25th to 75th percentile), whiskers show the range of the data (extending ≤1.5 × IQR) and dots indicate outliers. The Wilcoxon rank-sum test (Mann–Whitney *U* test) was used for group comparisons. The proportion of infants consuming foods among (D) breastfed and (E) weaned infants stratified by infant age. The number of samples per time point varies, explaining the nonlinear fluctuations in food consumption; specifically, the decrease in the proportion of infants using formula (month 5), cow milk (months 4 and 6), wheat (month 4), meats (month 12), and increase in the proportion of infants who were breastfed (month 6). Because of RCT-posed limitations on dairy proteins, cow milk and cow meat, their intakes were limited during the first 9 mo, with 1 noncompliant subject at months 3, 5, 7, and 8. RCT, randomized controlled trial.

The infants’ dietary pattern followed the typical progression from exclusive and partial breastfeeding to a diet primarily composed of solid foods by the end of the first year as reflected in changes in macronutrient intakes (Figure 2A–E). Most infants consumed infant formula during weaning. The weaning period refers to the transition from human milk to other food sources, including formula, solid foods, or both. Infants who were fully weaned, that is, not consuming any human milk, showed significantly higher formula consumption (Figure 2D and E), and energy intake from formula (Wilcoxon, *P* < 0.001; Supplemental Table 2) compared with breastfed. Overall breastfeeding rates decreased from 98% at 3 mo to 88% at 6 mo, 63% at 9 mo, and 41% at 12 mo.

Energy intake between infants who were breastfed and those who were weaned showed no significant differences at any age (*P* > 0.05) (Supplemental Table 2). Significant variations were observed in the proportion of energy (*E*%) from protein (Figure 2A) and fat (Figure 2B) on the basis of breastfeeding status. Overall, as infants transitioned from breastfeeding to formula feeding and began consuming complementary foods, there was a shift toward increased energy derived from proteins and carbohydrates across all age groups, with a corresponding decrease in energy from fats. The intake differences in protein and fatty acids between infants who were breastfed and weaned primarily resulted from the nutritional differences of mature human milk, which is richer in fats, and the infant formulas used, which had a higher protein content.

**FIGURE 3:**
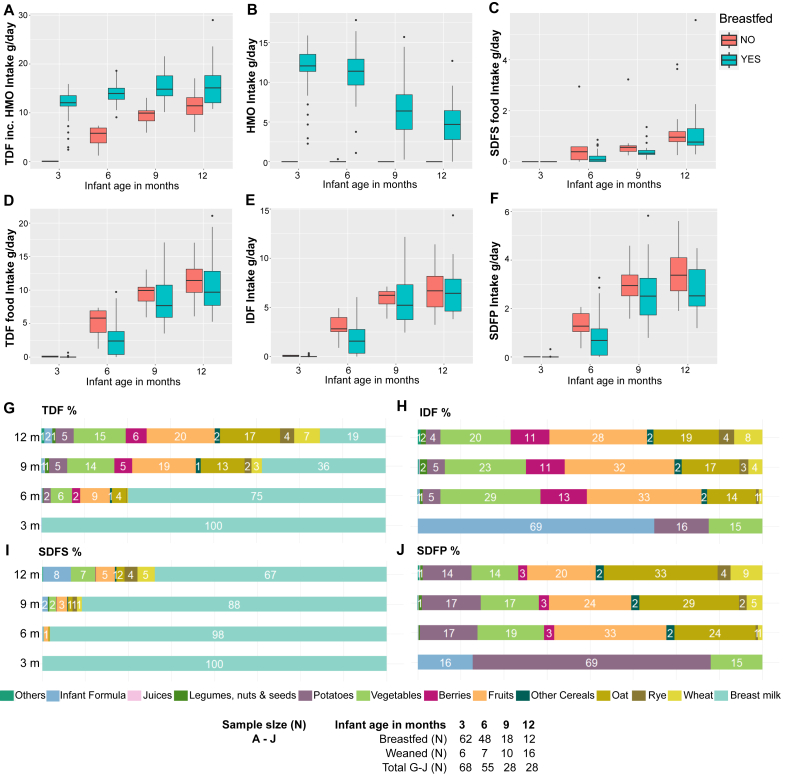
Fiber Intake and their food sources in infancy. (A) TDF (*P* < 0.05 across all months) and (B–F) fiber fraction intakes (g/d) are shown stratified by breastfeeding status (*P* < 0.05 indicated with ∗ below) and their food sources presented (% of total fiber/fiber fraction, TDF%). Fiber fractions include SDFS derived from breast milk only, marked as human milk oligosaccharide (HMO, B, ∗ across all months), SDFS derived from complementary food sources (not significant) (C), Combined TDF from complementary foods (∗ month 6) (D), IDF (E, ∗ month 6), and SDFP (∗ month 6). For boxplots, midlines represent the median, boxes the IQR (25th to 75th percentile), whiskers show the range of the data (1.5 × IQR), and dots any outliers. The Wilcoxon rank-sum test (Mann–Whitney *U* test) was used for group comparisons (G–J) presents all food sources of fiber (breast milk and complementary foods) during first year of life within the cohort. Percentages below 1% are not shown. The study formulas were not considered as fiber supplemented but contained minor concentrations of SDFP and IDF. The SDFP% and IDF% intake from formula at 3 mo of age is based on 33 infants of whom 30 were infants who were partially breastfed. The formula-related fiber accounts for under 1% of total TDF (Figure 3G), but because of the near complete absence of food-related fiber at 3 mo of age, as only 3 infants had started solid foods, resulted in seemingly inflated intakes of formula-derived SDFP (16%) and IDF (69%) fiber. Despite the higher formula *E*% intake at 6 mo of age, formula-related fibers present under 1% of total fiber 6 mo onward when solid foods are consumed by all formula feeders at month 6 (42 infants). IDF, insoluble dietary fiber; SDFP, dietary fiber soluble in water but insoluble in 78% aqueous ethanol; SDFS dietary fiber soluble in both water and 78% aqueous ethanol; TDF, total dietary fiber.

Overall, infants who were breastfed had higher absolute (g) (Figure 3A, Supplemental Table 2) and energy adjusted (g/MJ) intakes of TDF (Supplemental Table 2) at all age points compared with infants who were weaned, due to HMO intake (Figure 3B), uniquely available from human milk (*P* < 0.0001). The lower median intake of total SDFS fiber (including HMOs), and TDF among infants who were fed formula was fully explained by the absence of HMOs in formula and the negligible (under 1%) contribution of formula-derived SDFP and IDF intakes to TDF intakes at any month (TDF Infant formula, Supplemental Table 2).

The earliest introduction to complementary foods among both infants who were breastfed and weaned was at the age of 3 mo, in which case potatoes and vegetables constituted the sole complementary food sources of fiber (Supplemental Table 2). Consequently, absolute and energy adjusted intakes of complementary food derived fiber fractions of SDFS, IDF, and SDFP and total complementary food derived fiber increased among all ≤9 mo of age before stabilizing (Figure 3C–F, Supplemental Table 2). Fiber food source differences were minor, and in general complementary food fiber (Supplemental Table 2) intakes reached the same levels independent of breastfeeding status, even though earlier cessation of breastfeeding led to earlier introduction of most food groups (Figure 2D and E). As an exception, the complementary food fiber intake was higher among infants who were weaned at month 6 when only 85% of the infants who were breastfed had started solid foods as opposed to 100% of the weaned. This difference was attributed to 1.2 g/d higher median intake of IDF fraction (breastfed 1.6 g/d, IQR: 0.3–2.8; weaned 2.8 g/d, IQR: 2.5–4 g/d; *P* = 0.04) and higher median consumption of vegetables (*P* = 0.024), the main source of IDF, among weaned at month 6 (Supplemental Table 2). Additionally, the median fiber intake from vegetables was significantly higher in infants who were weaned at 9 mo (*P* = 0.008), and from oats at month 12 (*P* = 0.020); yet this did not result in a significantly higher intake of complementary food fibers during the corresponding months (Supplemental Table 2).

Overall, most of the complementary food-related dietary fiber in the EDIA cohort at months 6–9 came from fruits (from 9% to 19%) followed equally by vegetables (5%–14%), and cereals (oats, rye, wheat, and other cereals, 6%–18%). At month 12, cereals became the most common complementary food-related source of fiber constituting 30% of all fiber intake, fruits being second (20%). Oats were the most prominent contributor of cereal fiber and the largest single source of complementary food derived fiber at months 6–12 (Supplemental Table 2).

### Microbiome alpha and beta diversities followed typical development in infancy

Breastfeeding was the most significant dietary factor shaping the gut microbial composition in early infancy (beta diversity, Bray–Curtis dissimilarity, PERMANOVA, FDR <0.05, months 6 and 9) (Supplemental Figure 2B), followed by infant age (Supplemental Figure 2A). SDFP fiber intake from complementary foods at month 12 was associated with the overall microbiome composition (PERMANOVA test month 12, *R*^2^ = 0.31, FDR-corrected *P* = 0.03). In contrast, no other complementary food-related variable or any of the food group start age showed a statistically significant association with microbiome composition.

Human milk and its fiber were associated with lower species diversity (Supplemental Figure 2C and D, alpha diversity), whereas complementary food-related TDF (Supplemental Figure 2E) and fiber fractions (IDF and SDFP) displayed an opposite significant association during core diet-transition period from month 3 to 9 (Table 1, Supplemental Table 3). Fibers from oats and other cereals, along with the introduction of fish and meats, associated with diversity during this period (Table 1).

**TABLE 1:** Main nutrition results of mixed effect linear regression models predicting Shannon α diversity during the transitional period from 3 to 9 mo.

Nutrition component	Samples included	β coefficient	95% CI	*P* value
Exclusive breastfeeding	0.5–6	0.13	[–0.07, 0.33]	0.214
SDFS total inc. HMO (g/MJ)	Transitional period	–0.11	[0.03, 0.19]	0.009∗∗
TDF complementary foods (g/MJ)	Transitional period	0.12	[0.02, 0.22]	0.021∗
IDF (g/MJ)	Transitional period	0.18	[0.02, 0.34]	0.027∗
SDFP (g/MJ)	Transitional period	0.41	[0.09, 0.73]	0.012∗
SDFS complementary foods (g/MJ)	Transitional period	0.15	[–0.47, 0.77]	0.620
FFG–other cereals (g/MJ)	Transitional period	0.0024	[0.0013, 0.0046]	0.026∗
FFG–oat (g/MJ)	Transitional period	0.00025	[–0.00001, 0.00051]	0.039∗
Start age–fish	Food start age	0.25	[0.09, 0.41]	0.001∗∗
Start age–meats	Food start age	0.20	[0.04, 0.36]	0.007∗∗

Abbreviations: CI, confidence interval; FFG, fiber food group; HMO, human milk oligosaccharides; IDF, insoluble dietary fiber; SDFP, dietary fiber soluble in water but insoluble in 78% aqueous ethanol; SDFS dietary fiber soluble in both water and 78% aqueous ethanol; TDF, total dietary fiber.

Each row shows a main nutrition variable being modeled as the main variable in the longitudinal regression model the results for energy adjusted fiber, fiber fractions, and main macronutrient models are shown using data from food records during the transitional period, including months 3, 6, and 9 (*n* = 150). In the case of food sources of fiber and start age–variables only significant results are shown. Start age–variables are based on all available food start age questionnaire months starting from 2 wk of age and continued at monthly intervals from months 1 to 9, concluding at 12 mo. Each model has been adjusted with birth mode, age, sex, breastfeeding status, formula use, recent antibiotic use, solid food start age, and subject ID as a random effect. Other cereals included corn, rice, buckwheat and millet. ∗*P* <0.05; ∗∗*P* <0.01.

### Numerous infant-specific bacterial trajectories directly associated with complementary foods and their fibers, and inversely with human milk-related components

**FIGURE 4:**
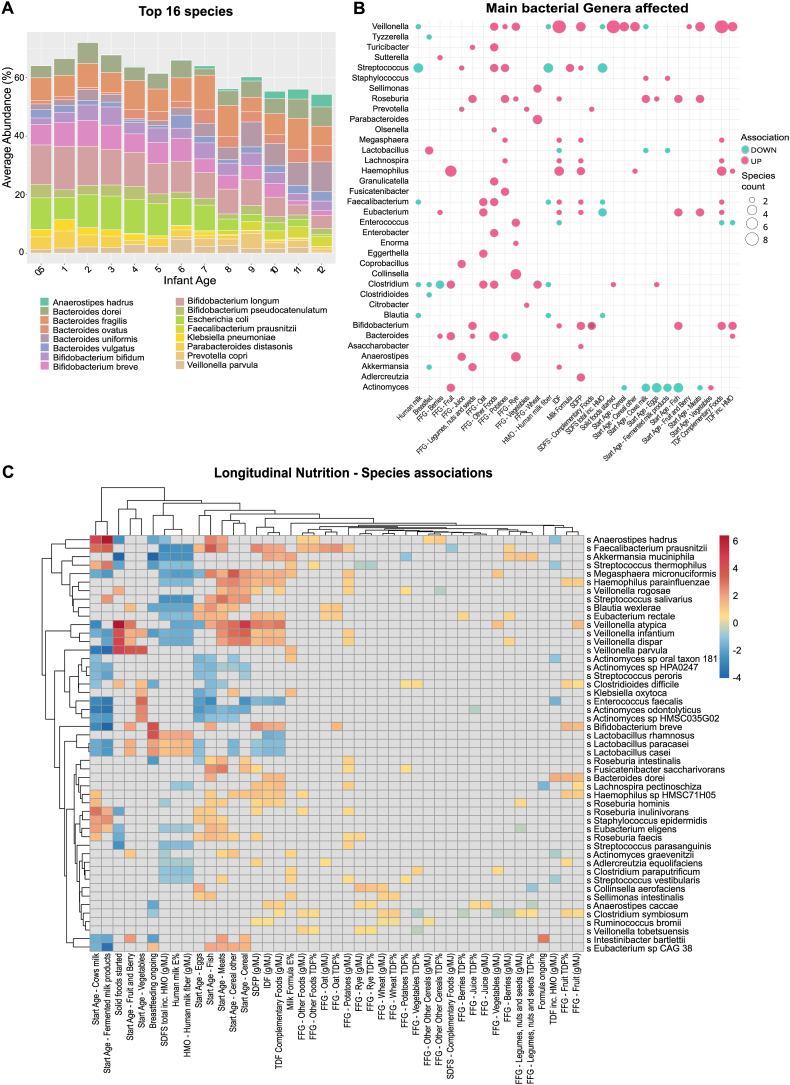
Longitudinal trends in bacterial taxonomy and their associations with infant diet. (A) The relative abundance of 16 most representative bacterial species in the EDIA cohort during infancy. The species were selected by picking the top 8 most abundant species, on average, in months 1, 3, 6, 9, and 12 among all infants, which amounted to a total of 16 distinct species. (B) Counts of significant (*Q*-value <0.10) direct and inverse nutrition-species associations per genera (counts >1 shown) from mixed effect linear models (see below section C). SDFS from complementary foods displayed both direct (*B. animalis*) and inverse (*B. longum* and *B. breve*) associations among *Bifidobacterium* species. Species association counts consist of the unique nonoverlapping associations based on all measurement categories per variable. (C) Multivariate association with mixed effect linear model (MaAsLin2) longitudinal analysis results for most shared species by different nutrition categories: Quantitative – Food record data (*n* = 179); Start Age (*n* = 480); Solid foods started/Breastfeeding/Formula ongoing (yes/no, *n* = 533). Microbial abundances were log-transformed, and the analyses were adjusted for age, breastfeeding, formula use, sex, solid food start, recent antibiotic use, and subject-specific random effects. Each square represents the effect strength β/SD of a linear regression model, analyzed between the respective dietary component and bacterial species. The significance threshold was FDR-corrected *P* < 0.25. Gray boxes indicate no reported association for a given combination. The heatmap shows the most representable measurement category for each nutrition variable; the selection was based on the energy or absolute measurement category that had the highest number of species associations along with the lowest average FDR-corrected *P* among the shared species associations between the measurement categories. Namely, all different fiber fractions and main macronutrients are presented as g/MJ and *E*%, respectively. FFG associations display both TDF% and g/MJ categories because of multiple unique associations appearing in each category and, therefore, neither category alone was equally representative. EDIA, Early Dietary Intervention and Later Signs of Beta-Cell Autoimmunity; FDR, false discovery rate; FFG, fiber food group; SDFS dietary fiber soluble in both water and 78% aqueous ethanol; TDF, total dietary fiber.

Our analysis of the EDIA cohort identified 528 unique bacterial species, with the average number of species per sample increasing with age, reflecting the statistically significant rise in Shannon diversity over time (Supplemental Figure 2C). The top 16 species accounted for 55%–72% of total bacterial abundance, transitioning from early dominance of Bifidobacterium (21%–25%) and Bacteroides (20%) to a more diverse Bacteroides-dominated (36%) composition by 12 mo (Figure 4A).

Our nutritional analyses yielded a total of 550 associations, both direct (387) and inverse (163) (Table 2, Supplemental Table 4), involving 5–37 species per listed nutrition category Table 2). Expectedly, numerous nutritional associations across different main nutrition categories overlapped, resulting in 136 distinct species among associations, with 75 species showing both direct and inverse correlations with nutritional components. Among these, 61 associations were unique to longitudinal analyses and absent in cross-sectional analyses at 3, 6, 9, and 12 mo. Figure 4B presents a summary count of nutrition-species associations, showing the impact (FDR-corrected *P* < 0.10) on 36 of 60 total genera affected (FDR-corrected *P* < 0.25), with fibers influencing 39 total and 33 of the main 36 affected genera.

**TABLE 2:** Counts of unique direct (*β+*) and inverse (*β*–) nutrition-species associations.

Nutrition category	β+	β–	Total count^1^	Total unique count within category^2^
Breast milk and HMO (amount)	4	21	25	
Milk-based formula (amount)	17	3	20	
Distinct FFG and complementary food fiber	96	51	51	
Complementary food fiber fractions total	70	17	87	63 (β + 51/β – 12)
○ *TDF complementary foods*	19	5	24	
○ *IDF*	19	4	23	
○ *SDFP*	24	4	28	
○ *SDFS complementary foods*	8	4	12	
FFG total	147	29	176	116 (β + 90/β – 26)
○ *FFG—berries*	11	3	14	
○ *FFG—fruit*	15	0	15	
○ *FFG—juices*	8	2	10	
○ *FFG—legumes, nuts, and seeds*	9	6	15	
○ *FFG—oats*	16	1	17	
○ *FFG—other cereals: corn, rice, buckwheat, millet*	6	2	8	
○ *FFG—other dietary fiber*	24	2	26	
○ *FFG—potatoes*	15	5	20	
○ *FFG—rye*	14	3	17	
○ *FFG—vegetables*	13	5	18	
○ *FFG—wheat*	16	0	16	
Food group start age	141	85	226	86 (β + 56/β −30)
○ *Start age—cereal other: corn, rice, buckwheat, millet*	17	8	25	
○ *Start age —cereal: oat, wheat, rye, barley*	13	6	19	
○ *Start age—cow milk*	14	18	32	
○ *Start age—eggs*	13	15	28	
○ *Start Age—fermented milk products*	16	16	32	
○ *Start age—fish*	25	12	37	
○ *Start age—fruit and berries*	10	2	12	
○ *Start age—meats*	26	6	32	
○ *Start age—vegetables*	7	2	9	
Breastfed (categorical)	6	17	23	
Exclusively breastfed (categorical)	1	4	5	
Solids started	8	8	16	
Total associations	387	163	550	

Abbreviations: FFG, fiber food group; HMO, human milk oligosaccharides; IDF, insoluble dietary fiber; SDFP, dietary fiber soluble in water but insoluble in 78% aqueous ethanol; SDFS dietary fiber soluble in both water and 78% aqueous ethanol; TDF, total dietary fiber.

1 The total count consists of only the unique bacterial associations per given nutrition subcategory.

2 The total unique count for combined top category (if applicable) and does not therefore duplicate any overlapping findings from the tested separate measurement categories (g, g/MJ/TDF%, *E*%).

Breastfeeding-related variables largely correlated with the same species as complementary food fibers, but in the opposite direction (Figure 4C, Supplemental Table 4). Breastfeeding associated inversely with genera commonly present in later infant gut trajectories reflective of more complex and stable communities [4,5,24], including *Faecalibacterium prausnitznii (Q = 0.0004), Blautia wexlare (Q = 0.004), Roseburia intestinalis* (*Q* = 0.001), and *Akkermansia munichipila (Q = 0.0008)*. Human milk intake (*E*%) correlated directly with bacteria present in human milk including *Lactobacillus* species L. *casei* (β,1.43, *Q* = 0.14), *L. paracasei* (β,1.32, *Q* = 0.13) and *L. rhamnosus* (β,1.87, *Q* = 0.21), which can utilize lactose and HMO byproducts [14]. However, exclusive breastfeeding alone, rather than human milk *E*%, was associated with *Bifidobacterium bifidum* (β,2.47, *Q* = 0.07) and *B. longum* (β,2.26, *Q* = 0.15; Supplemental Table 4). These species are recognized as infant-specific subspecies capable for primary degradation of HMOs and showed time-dependent decline in relative abundance within the cohort (Figure 4A).

Furthermore, distinct bacteria associated with nutritional components at each cross-section (3,6,9,12 mo) reflecting the shifts in food consumption and maturation trajectory typical bacterial species (Figure 4C). Cross-sectional species findings at the age of 6 mo displayed most nutrition associations with a total of 108 to 37 unique species, co-occurring with the first introductions of most food groups by this age among 58 infants.

### Distinct fiber food groups of the complementary diet had distinct associations to diverse gut bacterial species

In total 176 associations were found within different FFGs with some species-overlap between the distinct sources of fiber (for example, both fruit and oats directly associated with *Bifidobacterium breve*, Figure 4C). After accounting for this overlap 116 unique species level impacts remained (FFG; 90 × β+, 26 × β–; Table 2). Expectedly, FFG species-associations also overlapped partly with the findings from complementary food fiber fractions of IDF, SDFP, and SDFS – Complementary Food (87 total/63 total unique, Table 2). Therefore, the combined unique fiber-related associations totaled to 147 with 96 positive and 51 inverse associations to distinct species reflecting the vast impact of fibers on gut bacterial species, even after accounting for the redundancies of different fiber sources. Overall, complementary food fiber fractions, SDFP with 24 and IDF with 19 positive associations, shared equally significant associations to various mostly later trajectory species, albeit IDF with smaller effect sizes (β).

**FIGURE 5:**
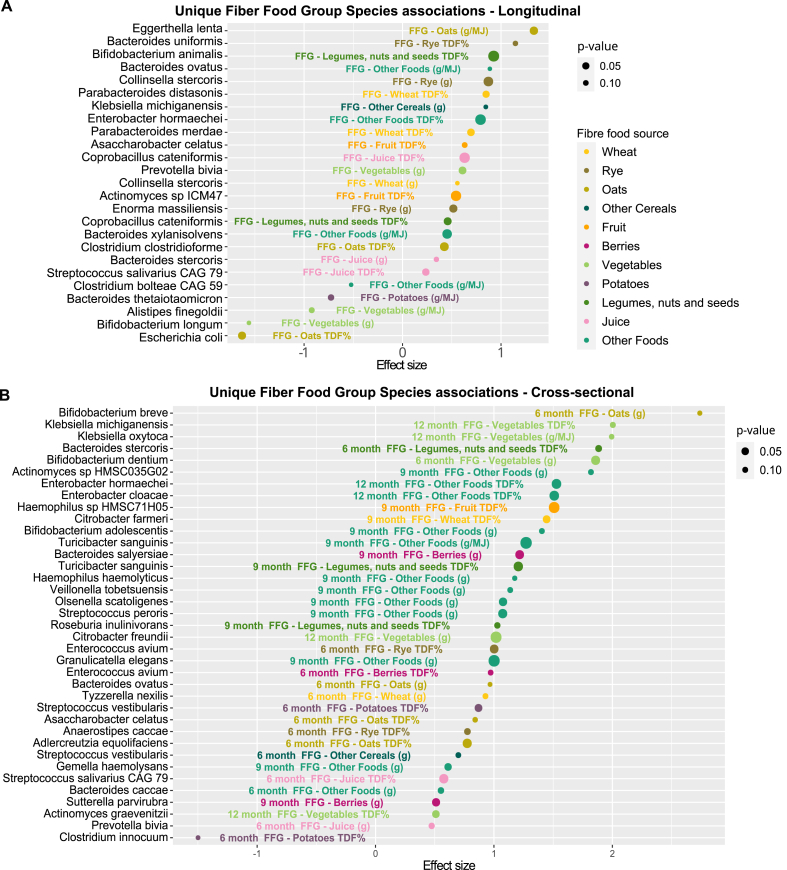
Multivariate association with mixed effect linear model (MaAsLin2) of (A) longitudinal (*n* = 179) and (B) cross-sectional (6 mo, *n* = 55; 9 mo *n* = 28; 12 mo *n* = 28) analysis results for unique FFG associations not uncovered by fiber fractions. After removing the species already visualized in heatmap Figure 4C, because of covarying and/or opposite longitudinal associations among other than fiber nutritional variables, 25 unique species remained. The significance threshold was set at FDR-corrected *P* < 0.15. Bacterial abundances were log-transformed, and the analyses were adjusted for age, breastfeeding, formula use, sex, solid food start, and recent antibiotic use, and subject-specific random effects were used in longitudinal models. FFG associations display the FFG species association with the lowest FDR-corrected *P* (*P* value) available from g, TDF% or g/MJ category. FDR, false discovery rate; FFG, fiber food group; TDF, total dietary fiber.

FFGs displayed associations to 34 bacterial genera, *Bacteroides* species being most numerous among positive associations. Complementary fiber food fractions (IDF, SDFP, and SDFS complementary foods) were associated with species in 27 genera, including, for example, several members of *Streptococcus* and *Veillonella* species among the direct associations. The most significant (FDR-corrected *P* < 0.10) shared directly associating species among FFG and food fiber belonged to genera *Streptococcus* (5 spp.), *Bifidobacterium* (5 spp.), *Veillonella* (4 spp.), *Roseburia* (3 spp.), and *Bacteroides* (3 spp.) (Figure 4C), whereas *Clostridium* species showed both positive and inverse associations. These trends were species-specific. For example, among *Roseburia, R. inulivorans* had most significant correlations to later-introduced “Other Dietary Fiber” (*Q* = 0.06)*, R. Faecis* to rye (arabinoxylans, *Q* = 0.007), whereas *R. intestinalis* only associated with earlier introduced potatoes (starches, *Q* = 0.09). Among these findings 51 associations remained unique to FFGs (Figure 5A and B, FDR-corrected *P* < 0.15), whereas only 8 were unique to complementary food-related fiber fractions.

In turn, cross-sectional analyses revealed that the introduction of a novel dietary source of fiber associated with the diversification of the microbiome revealing unique fiber-species-specific temporal patterns reflecting the maturity of the microbiome (Figure 5B, Supplemental Table 4).

### Complementary diet caused a contrasting shift in breastfeeding associated fecal metabolites

**FIGURE 6:**
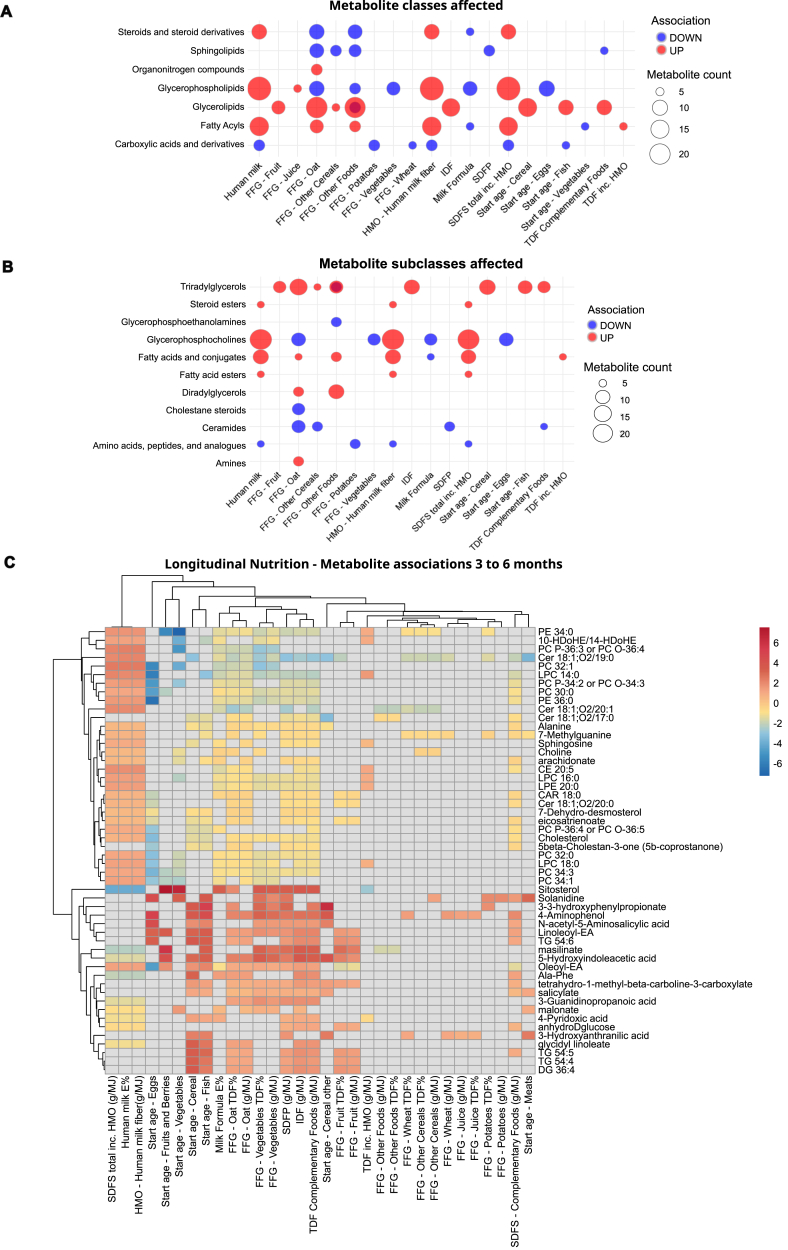
Longitudinal trends in fecal metabolites and their associations with infant diet until 6 mo of age. (A) An overview of nutrition–metabolite associations from mixed effect linear models (FDR-corrected *P* < 0.10). The results are summarized on the basis of the count per metabolite class (counts >3 shown). The start of solid foods displayed both direct and inverse associations among *Carboxylic acids and derivatives* class. FFG – metabolite association counts consist of the unique nonoverlapping associations based on all measurement categories (TDF%, g/MJ, or g) because of multiple unique associations appearing in each category and, therefore, neither category alone was equally representative. (B) An overview of nutrition–metabolite associations from mixed effect linear models (FDR-corrected *P* < 0.10, see below section C). The results are summarized on the basis of the count per metabolite subclass. The start of solid foods displayed both direct and inverse associations among *Aminoacids, peptides and analogs* subclasses. Metabolite association counts consist of unique nonoverlapping associations based on all measurement categories per variable. (C) Multivariate association with mixed effect linear model (MaAsLin2) longitudinal analysis results for most shared metabolites by different nutrition categories: Quantitative - Food record data (*n* = 58); Start Age (*n* = 125); Solid foods started/Breastfeeding/Formula ongoing (yes/no, *n* = 125). Metabolite abundances were log-transformed, and the analyses were adjusted for age, breastfeeding, formula use, sex, solid food start, recent antibiotic use, and subject-specific random effects. Each square represents the effect strength β/SD of a linear regression model, run between the respective dietary component and bacterial species. The significance threshold was FDR-corrected *P* < 0.25. Gray boxes indicate no reported association for a given combination. The heatmap shows the most representable measurement category for each nutrition variable; the selection was based on the energy or absolute measurement category that had the highest number of species associations along with the lowest average FDR-corrected *P* value among the shared species associations between the measurement categories. Namely, all different fiber fractions are presented as g/MJ. FFG associations display both TDF% and g/MJ categories because of multiple unique associations appearing in each category and, therefore, neither category alone was equally representative. TDF%, describing the proportion of the complementary food fiber source from the total complementary food fiber intakes among FFGs. FDR, false discovery rate; FFG, fiber food group; TDF, total dietary fiber.

In total, during the first year there were 797 identified metabolites within the subset of 33 infants in the EDIA cohort, monitored longitudinally until month 6 (Table 3, Supplemental Table 5). All the infants included were at least partially breastfed at the time of the fecal metabolite sampling. The overall effects of human milk and food fibers were clearly contrasting for each category (Figure 6A–C). Overall human milk and its fiber were mainly associated with increased abundance of metabolites (93 × β+; 36 × β–) including glycerophosphocholines (31 × β+) and the subclass of “fatty acids and conjugates” (17 × β+) being the most abundant among direct associations.

**TABLE 3:** Counts of unique direct (*β+*) and inverse (*β–*) nutrition–metabolite associations.

Nutrition category	β+	β–	Total count^1^	Total unique count within category^2^
Breast milk and HMO (amount)	93	36	129	
Milk-based formula (amount)	33	77	110	
Distinct FFG and complementary food fiber	108	97	205	
Complementary food fiber fractions total	142	131	273	(β + 142/β − 131)
○ *TDF complementary foods*	44	40	84	
○ *SDFP*	38	33	71	
○ *IDF*	43	36	79	
○ *SDFS complementary foods*	17	22	39	
FFG total	152	131	283	(β + 152/β − 131)
○ *FFG—berries*	2	0	2	
○ *FFG—fruit*	24	7	31	
○ *FFG—juices*	13	3	16	
○ *FFG—oats*	39	42	81	
○ *FFG—other cereals: corn, rice, buckwheat, millet*	10	10	20	
○ *FFG—other foods*	27	19	46	
○ *FFG–rye*	1	2	3	
○ *FFG–vegetables*	22	30	52	
○ *FFG–wheat*	6	8	14	
○ *FFG–legumes, nuts, and seeds*	4	2	6	
○ *FFG–potatoes*	4	8	12	
Start age	236	222	458	(β + 132/β − 156)
○ *Start age–cereal: oat, wheat, rye, barley*	66	41	107	
○ *Start age–cereal other: corn, rice, buckwheat, millet*	25	9	34	
○ *Start age–eggs*	29	57	86	
○ *Start age–fish*	60	41	101	
○ *Start age–fruits and berries*	16	11	27	
○ *Start age–meats*	24	11	35	
○*Start age–vegetables*	16	52	68	
Breastfed (categorial)	20	12	32	
Exclusively breastfed (categorial)	7	17	24	
Formula ongoing (categorial)	11	85	96	
Solids started	128	44	172	
Total associations	822	755	1577	

Abbreviations: FFG, fiber food group; HMO, human milk oligosaccharides; IDF, insoluble dietary fiber; SDFP, dietary fiber soluble in water but insoluble in 78% aqueous ethanol; SDFS dietary fiber soluble in both water and 78% aqueous ethanol; TDF, total dietary fiber.

1 The total count consists of only the unique metabolite associations per given nutrition subcategory.

2 The total unique count for combined top category (if applicable) and does not therefore duplicate any overlapping findings from the tested separate measurement categories (g/g/MJ/TDF%, *E*%).

In turn, human milk (9 × β–), FFGs (unique total 10 × β–) and complementary food-related fiber fractions (unique total 20 × β–) were associated with low levels of metabolite subclass “Amino acids, peptides, and analogues” (9 × β–), whereas formula milk (15 × β+) was associated with higher levels of the metabolites within that subclass (Supplemental Table 5).

Human milk and fibers from complementary foods displayed varied associations with immunomodulatory oxylipins such as hydroxydocosahexaenoic acid, hydroxyeicosapentaenoic acid, and hydroxyeicosatetraenoic acid, derived from essential fatty acids. In turn, human milk was directly associated with phosphatidylcholines (PCs) and their lysophosphatidylcholine (LPC) derivative(for example, LPC 16:1 β, 2.81 *Q* = 0.03; LPC 18:3 β, 1.26 *Q* = 0.03), whereas complementary food fibers showed inverse associations. Complementary food fibers or their introduction (Start Age) associated directly with various aromatic amino acid (AAA) derived metabolites including 5-hydroxyindoleacetic acid (5-HIAA), indole-3-propionate (I3P), and phenyllactate (Figure 6C, Supplemental Table 5).

Complementary food fiber fractions and their fiber sources associated most numerously to relatively higher abundance of diacylglycerols (DGs) and triacylglycerols (TGs) (TG: 18/30 × β+, DG; 5/10 × β+). Specifically, IDF, oats, fruits, and other dietary fiber (FFG – Other Foods) all displayed multiple associations to partly distinct DG and TG lipid species (8–15 × β+ each), many of which shared with fish (15 × β+). However, formula feeding, vegetable consumption or vegetable start age did not associate with TG/DG despite being part of the infant diet from the age of 4 mo. Although food fiber intake was associated with fecal loss of these dietary fats (TGs and DGs), the TG species present did not overlap with TGs positively associated with human milk, that is, the primary source of fat at month 6. In turn, vegetable consumption, in particular, was associated with lower levels of glycerophosphocholines (17 × β–) along with oats (16 × β+). Overall, the introduction of various food categories followed the effects of complementary food fibers (Figure 6A–C).

Overall, the metabolite profiles between different fiber types represented a relatively low number of unique associations; although the count for FFGs was 283 associations and for complementary food fibers 273 associations, the collective unique count for fiber categories resulted only in 108 direct and 97 inverse associations with metabolites. Ceramides (CER- sphingolipid class; 35 × β–), phosphocolines (PC – glycerophospholipid class; 37 × β–, 3 × β+) lipophosphocholines within (LPC – glycerophosphocholine subclass within glycerophospholipid class; 21 × β–, 2 × β+), and glycerophosphoethanolamines (LPE, glycerophospholipid class; 10 × β–) shared most associations with complementary food-related fibers and FFGs.

Most of the 55 direct associations unique to FFG in comparison to complementary food fibers, were to varying DG and TG species (23/50) or other close variations of the shared fiber metabolite species such as CER, PC, or LPC. Additionally, certain compounds demonstrated unique associations with fiber intake within specific FFG categories: N-methylserotonin (Juice and Other Cereals), I3P (Other cereals, Oats, and Wheat), 10-heptadecenoate (FFG – Other Foods), eicosapentaenoic acid ethyl ester (Rye).

## Discussion

Our longitudinal analysis shows that dietary fibers from complementary foods associate with previously described maturation patterns in infant gut microbiome [1,4,5,23,29] and changes in fecal metabolite profiles, during partial breastfeeding. We provide novel information on intake and food sources of fiber fractions, including previously overlooked resistant starch and short-chain NDOs from fruits, vegetables, and whole grains, using CODEX-compliant fiber values relevant for gut microbial effects during infancy [[32], [37]]. Plant-based complementary food fibers associated with 176 bacterial species, with 84% showing positive associations, coinciding with appearances of new species related to the maturation of the infant gut microbiome. In turn, human milk with its fiber associated with lower species diversity, inversely associating with genera and species common in late infancy, suggesting that they control the maturation rate, as previously described [4,5,24].

Our data suggests that introducing fiber-rich, plant-based foods support the early life microbiota during weaning [4,5,51]. Notably, novel fiber sources of oats, fruits, vegetables, and potatoes, commonly found in first spoon-fed purees, showed associations to infant type bacterial species from genera *Bifidobacterium, Bacteroides, Lactobacillus*, *Streptococcus, Enteroccoccus*, and *Veillonella* [9,[52], [53], [54]] with key roles in immunomodulation [11,52,55,56]. In contrast, a 7-wk RCT found that infant cereals with added sugar led to lower abundance of these infant type taxa compared with the low-sugar version, despite identical fiber content [57]. The correlations between SDFS fraction, primarily from fruits and vegetables, and microbial taxa, such as *Faecalibacterium prausnitzii*, resembled human milk effects [4,5], likely because of fibers such as fructo-oligosaccharides with bifidogenic effect [58,59]. Although breastfeeding remains the nutritional gold standard, these findings emphasize the importance of considering the impacts of first food choices on gut microbiome composition and maturation.

More mature gut microbiome communities appeared around 9 mo when complementary food fiber intake (7.7 g/d) surpassed HMO intake (6.4 g/d) in infants who were partially breastfed. These included plant-fiber fermenting species among *Clostridia*, and butyrate producers such as *F. prausnitzii* and species among *Roseburia, Anaerostipes*, and *Eubacterium* genera displayed late (9–12 mo) associations with all plant-based dietary fiber sources in a species-specific manner. The SDFP fraction, along with IDF, berries and oats, was associated with lactate producing *B. breve* and butyrate producing “weaning transition” taxa, such as *Roseburia inulivorans, Eubacterium rectale*, and *F. prausnitzii* [4,8,24,60]. This likely reflects known interspecies cross-feeding between primary degraders such as *Bifidobacteria, Lactobacilli,* and *Bacteroides* and butyrate producers, facilitating the taxa transition [52,61,62]. The strongest positive associations were with later-introduced foods such as meat, dairy, rye, and coincide with increased complex fiber and protein intake at 9 mo, known to alter gut pH and oxygen levels [52,54,63], fitting with previous findings [8,20,[64], [65], [66]]. Overall, our fiber–taxa associations represented key anaerobe taxa involved in community assembly, core metabolic functions, and colonization resistance against pathogens [8,24,60].

Given that the infant gut microbiome stabilizes close to adult-like composition by 3–5 y of age [4], early diet plays a key role in structuring microbial communities. Our results support the consensus that diverse fibers from complementary foods support a complex microbial ecosystem with potential health benefits [28,[67], [68], [69]]. Oats, rye, wheat, and potatoes were associated with 16, 14, 16, and 15 mostly unique bacterial species, respectively, suggesting a selective impact of unique fiber structures on bacterial communities. Even small amounts of fiber, such as a spoonful of mixed berries (12 g, fiber: 0.33–0.41 g/d), can have a notable impact in infancy, evidenced by the 14 distinct bacterial associations. Furthermore, diet may play a particularly potent role structuring metabolic capabilities of bacteria during weaning [33,[70], [71], [72]], because of increased transfer of genetic material between bacteria, related to dietary substrate metabolism [33]. For example, Bacteroides spp. and *B. ovatus*, *B. stercoris*, *B. caccae,* known for genetic versality, displayed equally significant associations with food fibers as well as fish and meat, suggesting adaptation to diverse dietary substrates [21].

We further investigated the impact of solid foods on the fecal metabolomes of 33 infants who continued partial breastfeeding after solid food introduction—a practice recommended by WHO [73]. Weaning was associated with metabolites relevant for immune and metabolic health, both diversifying metabolic pathways, as well as contributing to those influenced by human milk.

During weaning, AAA catabolite profiles are known to shift from HMO-adapted Bifidobacterium species producing lactic acids such as indole-3-lactic acid (ILA) to a broader range of bioactive AAA catabolites, driven by changes in microbial taxa and substrate availability [[74], [75], [76]]. Weaning diet, including the introduction of gluten-free cereals, was associated with increased phenyllactate whereas tryptophan-derived 5-hydroxyindoleacetic acid (5-HIAA) was associated with high-fiber foods such as oats and vegetables, and I3P linked to total complementary food fibers. These metabolites are known to enhance gut epithelial barrier function [77,78] and may explain a previous association between fruits, vegetables and oats, and reduced gut permeability and systemic inflammation in EDIA cohort infants [34,79]. Although weaning increases both fiber and protein intake, higher fiber reduces microbial protein catabolism and promotes more favorable AAA fermentation profiles [36,43], including greater production of ILA and indole-3-propionic acid [80]. Notably, the introduction of fruit, berries, and cereals was associated with increased 5-HIAA levels more strongly than typical protein (tryptophan) rich foods of fish, and 5-HIAA showed no association to eggs. AAA catabolites may offer protective roles against colitis and autoimmunity [81,82] and enhance insulin sensitivity because of lower systemic inflammation [77,83,84].

Weaning triggers a time-sensitive mechanism promoting microbially mediated immune tolerance, “weaning reaction” [40], and the introduction of allergenic foods such as nuts and eggs is, therefore, recommended during 4-to-6-mo window to reduce allergy risk [[85], [86], [87], [88], [89]]. Our results indicate that complementary fibers during early weaning were associated with broad shifts in immunomodulatory metabolites, including PCs, LPCs, and diverse fat and amino-acid derivatives (for example, indoles), that often inversely correlated with human milk effects. We observed changes in fecal metabolites previously linked to food allergy risk during infancy, such as bile acids, DGs, sphingolipids, ceramides, oxylipins, and plant polyphenols [81,[90], [91], [92], [93]]. Dietary fibers and associated compounds, interacting with or modulated by microbes, may modulate allergy risk [40,52], whereas disturbed microbiota maturation in itself is associated with increased pediatric allergic conditions, including asthma [27,94]. Our fecal microbiome and metabolomics data underscore the need to investigate the role of diet, the timing of fiber-rich plant food introduction and the gut microbiome in allergic sensitization.

This study has limitations. The EDIA cohort included infants with only certain HLA-haplotypes, which may have influenced infant gut microbiome assembly [95] potentially limiting the generalizability of the results. The associative nature of our study, potential collinearity, and reliance on compositional relative abundance data present challenges in interpreting results [23,96], though similarities in taxa–diet associations across intercontinental infant cohorts support the broader applicability of our findings [97]. The secretor phenotype influences human milk HMO-concentrations [51], but individuals in this cohort were not genotyped for FUT2 SNPs, which may impact the accuracy of HMO estimations.

In conclusion, our longitudinal study highlights the importance of introducing fiber-rich complementary foods during weaning to support the gradual gut microbiome maturation. We demonstrate fiber-species-specific and temporal patterns, showing that even small dietary variations displayed significant association to taxa and metabolites. A deeper understanding of the diet-gut microbe axis can allow microbiota-guided fiber recommendations to strategically support gut microbiome maturation, using microbiome milestones [4,5,23,24] as measurable outcomes in future dietary interventions.

## Author contributions

The authors’ responsibilities were as follows – MKL: conducted the formal analysis and wrote the original draft; MKL, SMV, TV: conceived the study; MKL, SMV, TV: curated data; MK, SMV: collected stool samples and patient information; TV and SMV: supervised the work; and all authors read, commented, and approved the manuscript.

## Funding

We gratefully acknowledge the support provided by the National Institute of Diabetes and Digestive and Kidney Diseases (NIDDK); National Institutes of Health (1DP3DK094338-01); the Academy of Finland Centre of Excellence in Molecular Systems Immunology and Physiology Research 2012-17 (250114); the Medical Research Funds, Tampere and Helsinki University Hospitals; and the startup funding from the Helsinki Institute of Life Science, University of Helsinki, which supported this research. The funders had no role in the design, analysis or writing of this article.

### Data availability

Metagenomic and metabolomic data described in the manuscript are made publicly and freely available without restriction at NCBI Sequence Read Archive (SRA: PRJNA821542) and at Metabolomics Workbench (https://doi.org/10.21228/M8C70Q), respectively. This paper does not report original code. Analysis software including quality control, taxonomic, and functional profilers is publicly available and referenced as appropriate.

## Conflict of interest

TV has received speaker honoraria from Nestle Nutrition Institute (NNI).
