# Supplementary material for: Associations between dietary fibers and gut microbiome composition in the EDIA longitudinal infant cohort
Source: Am J Clin Nutr. 2024 Nov 16;121(1):83–99. doi: 10.1016/j.ajcnut.2024.11.011 (PMC11747200; doi:10.1016/j.ajcnut.2024.11.011)
Supplement: Multimedia component 1 [file mmc1.pdf]

Associations between dietary fibers and gut microbiome composition in the EDIA longitudinal infant cohort, Lalli M. et al.,

Online Supplementary Figures

This file includes:

Supplementary Fig.1 Participant flowchart.

Supplementary Fig. 2 Bacterial species diversity and compositionality of the infant gut in association with nutrition.

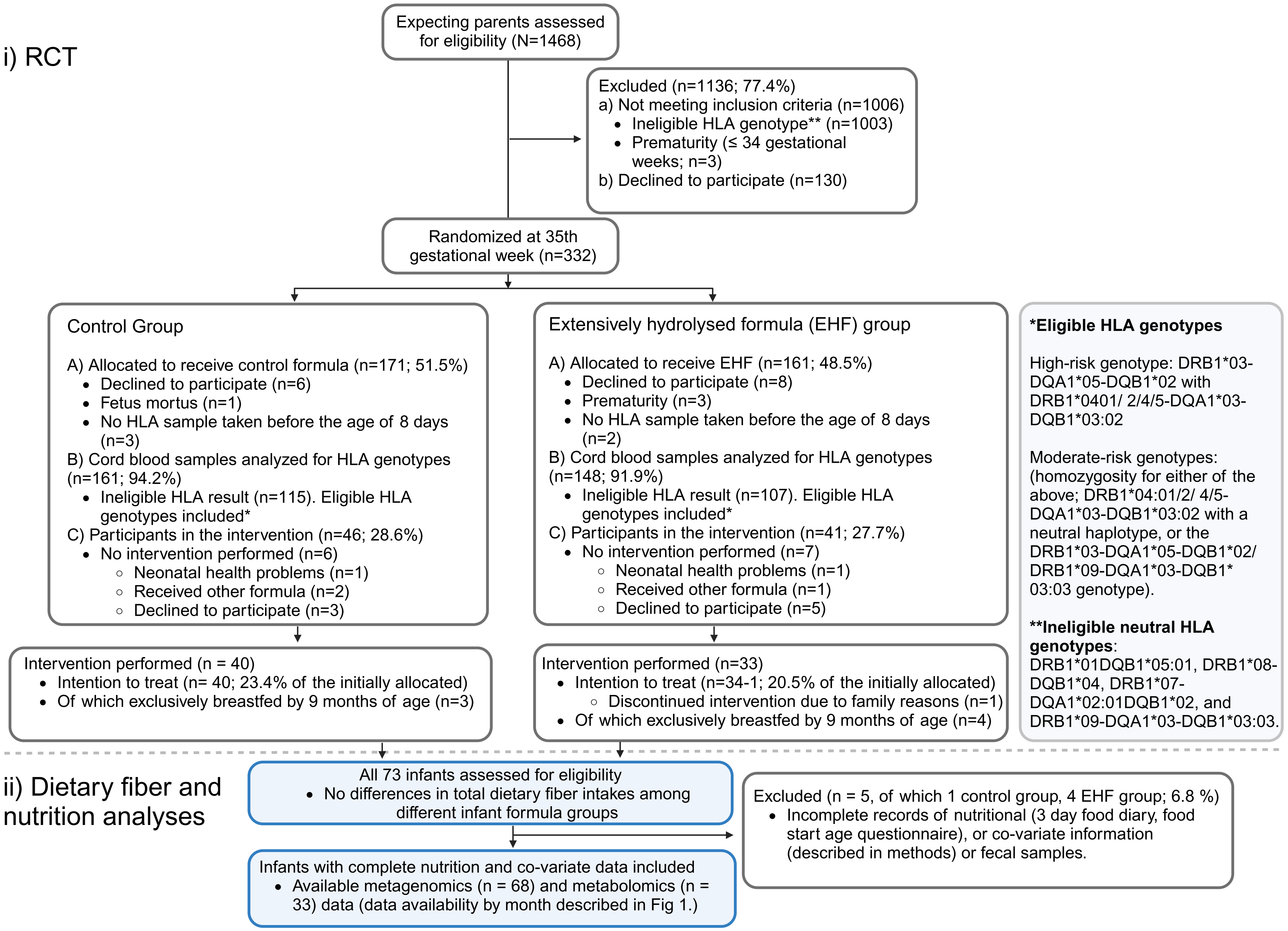

Supplementary Fig. 1 Participant flowchart outlining the participants included in the different analyses and criteria for exclusion.

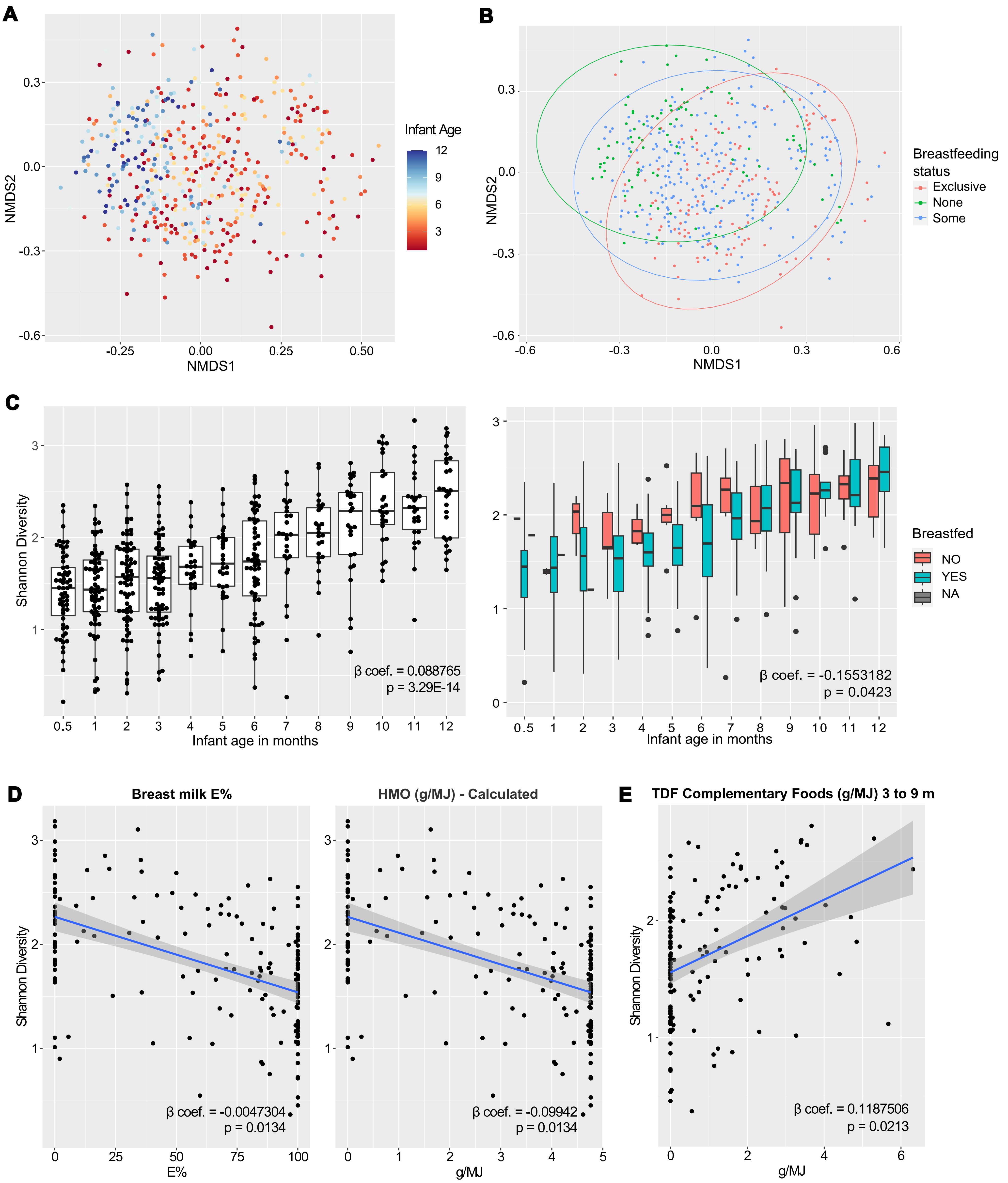

Supplementary Fig. 2. Bacterial species diversity and compositionality of the infant gut in association with nutrition. **(A-B)** Nonmetric multidimensional scaling (NMDS) ordination plot of gut microbiome compositional profiles of infants (n = 533) during the first year of life based on Bray-Curtis dissimilarities of species-level relative abundances from metagenomic data. Colors indicate **(A)** infant age (PERMANOVA longitudinal R2 =0.01209, p = 0.0001) and **(B)** breastfeeding status (exclusively breastfed, receiving none or some breast milk). **(C)** Microbiome alpha diversity (Shannon's diversity index) during the first year of life and stratified by breastfeeding status (n = 533). For boxplots, midlines represent the median, boxes the interquartile range (IQR, 25th to 75th percentile), whiskers show the range of the data (1.5*IQR), and dots indicate outliers. **(D)** Inverse associations between breast milk energy (E%) intake and energy adjusted HMO intake, and microbiome alpha diversity. Statistical significance was assessed by a linear model while controlling for the sampling month (months 3, 6, 9, and 12, n = 178) and the resulting beta-coefficient and p-values are reported. **(E)** An association with energy adjusted complementary food fiber intake and microbiome alpha diversity (Shannon's diversity) in data from months 3, 6, and 9, during core diet transition period (n = 150). Reported beta coefficients and p-values in C-E were adjusted for breastfeeding status, birth mode, age, sex, recent antibiotic use, solid food start, sampling month, and subject-specific random effects.
